# Can Programmed or Self-Selected Physical Activity Affect Physical Fitness of Adolescents?

**DOI:** 10.2478/hukin-2014-0097

**Published:** 2014-11-12

**Authors:** Cláudio F. Neto, Gabriel R. Neto, Adenilson T. Araújo, Maria S. C. Sousa, Juliana B. C. Sousa, Gilmário R. Batista, Victor M. M. R. Reis

**Affiliations:** 1 University of Trás-os-Montes and Alto Douro, Vila Real/Portugal.; 2 Federal University of Paraíba, João Pessoa, Brazil.; 3 Federal University of Rio de Janeiro, Rio de Janeiro, Brazil.; 4 Kinanthropometry and Human Development Laboratory, João Pessoa, Brazil.

**Keywords:** exercise, physical fitness, physical education, high school, youth

## Abstract

The aim of this study was to verify the effects of programmed and self-selected physical activities on the physical fitness of adolescents. High school adolescents, aged between 15 and 17 years, were divided into two experimental groups: a) a self-selected physical activity group (PAS) with 55 students (aged 15.7 ± 0.7 years), who performed physical activities with self-selected rhythm at the following sports: basketball, volleyball, handball, futsal and swimming; and b) a physical fitness training group (PFT) with 53 students (aged 16.0 ± 0.7 years), who performed programmed physical fitness exercises. Both types of activity were developed during 60 min classes. To assess physical fitness the PROESP-BR protocol was used. The statistical analysis was performed by repeated measures ANOVA. The measurements of pre and post-tests showed significantly different values after PFT in: 9 minute running test, medicine ball throw, horizontal jump, abdominal endurance, running speed and flexibility. After PAS differences were detected in abdominal endurance, agility, running speed and flexibility. The intervention with programmed physical activity promoted more changes in the physical abilities; however, in the self-selected program, agility was improved probably because of the practice of sports. Therefore, physical education teachers can use PFT to improve cardiorespiratory fitness and power of lower and upper limbs and PAS to improve agility of high school adolescents.

## Introduction

Morphological changes during adolescence are associated with several components of physical fitness (Vaeyens et al., 2005) which, according to the American College of Sports Medicine (ACSM, 2009; ACSM, 2011), is primarily determined by physical exercise and can be divided into the following physical fitness measures: aerobic power, flexibility, strength and skeletal muscle endurance.

Moreover, the American College of Sports Medicine (ACSM, 2004) also stated that children and adolescents should perform physical activity of high intensity at least three times a week from 10 to 20 min (twice or more per day may be more effective), and it should consist of gymnastics, plyometrics, jumping and endurance training of moderate intensity, on top of participation in sports that involve running and jumping (football, basketball, handball, volleyball, among others). According to the World Health Organization (WHO, 2010), children and adolescents should participate in moderate or vigorous intensity aerobic physical activities (such as running, hopping, skipping, jumping rope, swimming, dancing, and bicycling) that should comprise 60 or more minutes a day and high intensity physical activity should be included at least 3 days a week. In general, it appears that higher volumes or intensities of physical activity are likely to have greater benefit, but research in this area is still limited and the results are not convergent (Janssen, 2007; Tassitano et al., 2007).

Nevertheless, the programmed and systematized high intensity practice of physical activity seems to hamper exercise adherence, especially in school. This may be due to dissatisfaction and lack of pleasure in performing physical activity. Studies on intensity of self-selected exercises during physical activity and on the concurrent physiological changes are not novel. Dishman et al. (1994) indicated that people engaged in programmed physical activities preferred to practice various sports, due to self-selected intensities. Santos et al. (2009) compared the physiological and perceptual responses between normal weight, overweight and obese sedentary adult women, during walking at self-selected pace; and they found that the perceptual responses did not differ between the three groups.

Since only few studies have addressed the programmed vs. self-selected physical activity in the context of high-school physical education classes, performing such research seemed necessary. Therefore, the aim of this study was to verify the effects of programmed and self-selected physical activities on the physical fitness of adolescents. We hypothesized that practice of programmed physical activities had larger effects on physical performance compared to self-selected physical activity.

## Material and Methods

### Subjects

This study was approved by the Human *Research Ethics Committee* (HREC) of the Federal University of Paraíba – UFPB, Protocol No. CEP / CCS No. 0269, 2011 in accordance with the Declaration of Helsinki and resolution No. 196/96 of the National Health Council. A total of 108 high school students (1^st^, 2^nd^ and 3^rd^ year), aged between 15 and 17 years, from the Federal Institute of Education, Science and Technology of Paraíba (IFPB) / João Pessoa, Brazil, participated in the study. All of them were pubescent and post-pubescent, according to Tanner Stages of Sexual Development (1991). The sample comprised two randomly assigned experimental groups: a) a self-selected physical activity group (PAS) with 55 students (aged 15.7 ± 0.7 years), 27 males and 28 females, who performed self-selected physical activities and b) a physical fitness training group (PFT), with 53 students (aged 16.0 ± 0.7 years), 30 males and 23 females, who performed programmed physical training according to WHO (2010) recommendations. Inclusion criteria encompassed the following aspects: being regularly enrolled in the IFPB, participating in physical education classes, being free of infectious diseases and being authorized by the parents to take part in the research. After having the parental consent form signed and medical clearance, students were enrolled in the study. Subjects who, during the 16 week intervention, acquired any skeletal muscle injury or had attendance lower than 75% were excluded from the experiment.

### Study Design

After explaining the procedures, students were invited to participate in the study, being randomly assigned to develop two types of activities: a) self-selected physical activity, which was performed three times per week for 60 min and comprised of 20–25 min stretching as a warm up, dynamic joint movements followed by various sports chosen by the students (basketball, handball, volleyball, swimming and futsal). From the selected sports, competitive recreational games were applied and they lasted between 25 and 35 minutes. During the games, each student was replaced when showed either low physical performance (fatigue or continuous error) or psychological stress (unfair attitude). Finally, the students had 5 to 10 min of relaxation activities such as stretching, relaxation through static sound stimuli (sounds of nature) or just walking slowly, as decided by the group; b) programmed physical activity (fitness) was performed with the intensity level of 70 to 80% of the maximum heart rate (220-age) (Fox et al., 1971), following the recommended levels of physical activity for children and young people (WHO, 2010), performed for 60 min, three times per week. Each session comprised of 20 to 30 min of step up and down exercise and/or walking and jogging in the continuous and interval methods, plus 20–30 min of dynamic and static strength exercises, dynamic and static flexibility in circuit and interval methods. Lastly, the cooling-down phase lasted about 5 to 10 min of relaxation with activities of static stretching (10–30 s per movement).

### Physical fitness assessment

The PROESP-BR battery test described by Gaya and Silva (2007) was applied taking into consideration its common use in Brazilian high schools and little equipment needed. This battery comprises seven tests: a 9 min race for cardiorespiratory capacity (9MIN), a 1 min abdominal test for abdominal endurance (LMEabd), Sit and Reach for flexibility (FLEXw/b), a 20 m running test for speed (SP20m), a horizontal jump test for lower limb explosive power (LLEP), a medicine ball throwing test for upper limb explosive power (ULEP) and agility with the square test (AGILst). The subjects performed the tests before and after the 16 weeks intervention.

### Statistical Analysis

After checking data normality with the Kolmogorov-Smirnov and homogeneity by the Levene’s test, repeated measures ANOVA was applied to analyze the effect of time (pre-post ×), group (PAS × PFT), and the interaction effect (group × time). Additionally, a t test was run within groups with percentage variation (Δ%) and to estimate the effect size (η^2^), values greater than 0.20, 0.50, 0.80 and 1.30 represented small, medium, high and very high end, respectively (Cohen, 1988). The level of significance was set at p <0.05 and all statistical analyses were carried out using SPSS statistical software package version 20.0 (SPSS Inc., Chicago, IL).

## Results

The analysis of effect of time on the physical performance variables (Table 1) indicated that the majority of them presented significant changes, being the effect size medium in the 9MIN (p = 0.001; η^2^ = 0.50), small in the LMEabd (p = 0.001; η^2^ = 0.40) and FLEXw/b (p = 0.001; η^2^ = 0.40) tests, and were non-significant in SP20m (p = 0.001; η^2^ = 0.10) and LLEP (p = 0.004; η^2^ = 0.07) tests. The effect of time was not observed in the ULEP test nor in the AGILst test. As to the group effect, it was non-significant and only the 9MIN (p = 0.015, η^2^ = 0.05) and ULEP (p = 0.049; η^2^ = 0.03) variables presented significant changes. Other variables that were not sensible to the group effect were the LLEP, LMEabd, AGILst, SP20m and FLEXw/b tests. The analysis of interactive effect (time × group) proved significant for two variables: the 9MIN with small (p = 0.001; η^2^ = 0.40) and to RMLabd with trivial (p = 0.019; η^2^ = 0.05). There was no interactive effect for the ULEP, LLEP, AGILst, SP20m and FLEXw/b tests.

As can be inferred from Figure 3, in each group the observed *actual* TET proves to be *significantly shorter* than the predicted TET *only after sleep* (EME_[Retest 1]_: *t* (11) = −3.901, *p*_[one-tailed]_ = .001, *d* = 1.13; MEM_[Retest 2]_: *t* (11) = −5.019, *p*_[one-tailed]_ < .001, *d* = 1.41). Also, in the EME-group this sleep-induced advantage of actual over estimated TET-performance appears to be preserved during the The comparisons of pre vs. post-test measures in the various physical fitness tests (Table 2) presented significantly different values for the 9MIN (t=−13,257, p=0,001, Δ=25%), ULEP (t=−2,403, p=0,020, Δ%=6), LLEP (t=−2,524, p=0,015, Δ=13%), LMEabd (t=−7,205, p=0,001, Δ=27%), SP20m (t=4,367, p=0,001, Δ= −5%) and FLEXw/b test (t=−6,451, p=0,001, Δ=30%) for the PFT. For the PAS the values of LMEabd were different: (t=−4,138, p=0,001, Δ=14%), AGILst test (t=2,846, p=0,006, Δ= −4,3%), SP20m (t=2,250, p=0,029, Δ= −2,5%) and FLEXw/b test (t=−4,822, p=0,001, Δ=19%). Regarding the intra-group correlation coefficient, it was observed that the results were considered satisfactory (Table 2), indicating that the variation due to variability between individuals or problems in the administration of the test was small.

## Discussion

This study examined the effect of programmed physical fitness training (PFT) vs. self-selected physical activity (PAS) on adolescents’ physical fitness. We hypothesized that practice of programmed physical activities had larger effects on physical fitness compared to self-selected physical activity.

The main finding of the present study was that the PFT improved cardiorespiratory fitness and power of lower and upper limbs while PAS improved only agility of the students. Thus, our hypothesis was partially confirmed. Larger differences (as given by the relative variation, Δ%**)** between the administration of the two programs were observed in the cardiorespiratory fitness and in abdominal endurance. It is noteworthy that the self-selected practice of various sports (including game sports) was not able to promote cardiorespiratory adaptations. Hence, this may be the physical fitness component which requires extra engagement other than typical physical education classes. The changes observed in the remaining variables were less prominent and (or) may be mostly due to growth and maturation phenomena, despite the type of the program applied. Though, the magnitude of the changes may be influenced by hormonal release, which in turn may depend on the amount of physical activity (Mota, 2000).

To the best of our knowledge, the present study was the first to compare these two types of physical activity (self-selected vs. physical fitness training) within the school daily environment. Araujo and Oliveira (2008) previously analyzed a considerable sample of elementary students aged between 10 and 14 and found significant changes in some of the physical fitness variables tested in the current study which were consistent with the growth & maturation hypothesis. The study by Levandovski et al. (2007) investigated physical and motor tests, in a sample of 15 school futsal players aged 16–17 years and observed 31,27 reps in arm flexion, 37,53 reps in abdominal endurance, 1,71 m in the horizontal jump, 6,47 s in the agility test, and 1,888 m in a 12 min race. These values are close to those found in the present study which confirms that the profile of our subjects was similar to those typically found in Brazilian schools at this age group. In addition, the results of the boys and girls in the current study were within the standards established for the battery PROESP-BR (Gaya and Silva, 2007). Sabia et al. (2004) went further in an ecological study by comparing a program of aerobic exercises with another program including anaerobic exercises. They found that both, performed three times per week during 16 weeks, 20 to 40 min per session, significantly increased fitness in obese adolescents. A small-sample (22 high school adolescents) was analyzed by Willms and Fachineto (2011) and they concluded that physical activity during physical education classes can contribute to the health of school students by enabling various adaptations in physiological measures.

Freitas et al. (2010) conducted a study which may be considered to be the most comparable to our study. They verified the effect of programmed physical activity on physical fitness tests in adolescents at school during a period of 1 year. The study covered a total of 383 students aged between 10 and 15 years who were divided into two groups: physical activity program vs. typical physical education classes. Sit-and-reach testing was carried out, muscular endurance and cardiorespiratory fitness were assessed. The authors concluded that there was no difference between the groups, although there was a general improvement in strength and endurance after the intervention.

In summary, the present study showed differences between the two interventions regarding cardiorespiratory fitness in both genders, with higher values in the group that performed the prescribed exercise. Furthermore, in both groups and genders, applying programmed or self-selected activities, strength, endurance, speed and flexibility values increased. Nevertheless, it was perceived that the fitness training program was more effective, for both genders, since it was able to produce changes in more variables. These results recommend that the program of physical activities in high school should be diverse, stimulating both the engagement in fitness training and that in sports. Therefore, it is our opinion that high school syllabuses should be designed as to address the various types of physical activity. Poorly structured physical activity for these age groups can result in injuries, fractures, osteochondrosis, and menstrual dysfunction (Alves and Lima, 2008).

### Practical Applications

We concluded that the efficacy of both program was not significantly different. However, the programmed physical activity indicated to cause more changes in physical fitness. Thus, it may be useful for physical education teachers to include physical fitness exercises in every class in order to enhance physical abilities of students (strength, flexibility, speed, agility, flexibility and cardiorespiratory endurance) that need to be stimulated during adolescence.

## Figures and Tables

**Table 1:** Multivariate analysis of physical fitness tests (N = 108).

	**9MIN_(m)_**		**LMEabd_(rep)_**		**FLEXw/b_(cm)_**		**SP20m_(s)_**	
**Statistical Effect**	F	*post hoc*	F	*post hoc*	F/p-value	*post hoc*	F/p-value	*post hoc*

**Time (T)**	100.43^*^	Pre≠Post	65.26^*^	Pré≠Pós	64.68^*^	Pré≠Pós	18.00^*^	Pré≠Pós
**Group (G)**	6.13^*^	PFT≠PAS	0.11	-	0.00	-	0.26	-
**Interaction**	77.50^*^	-	5.63^*^	-	2.69	-	1.21	-

	**LLEP_(rep)_**		**ULEP_(rep)_**		**AGILst_(s)_**			

**Statistical Effect**	F	*post hoc*	F	*post hoc*	F	*post hoc*		
**Time (T)**	8.73^*^	Pre≠Post	3.69	-	1.378	-		
**Group (G)**	0.19	-	3.97^*^	PFT≠PAS	0.175	-		
**Interaction**	0.00	-	0.38	-	0.175	-		

(*) significant difference between the pre and post-test (p<0.05);

PFT = physical fitness training; PAS = self-selected physical activity; 9MIN = cardio-respiratory (9 min); LMEabd = abdominal endurance; FLEXw/b = flexibility (sit-and-reach); SP20m = 20 m running; LLEP = horizontal jump test; ULEP = medicine ball throwing; AGILst = agility.

**Table 2:** Descriptive values comparisons between moments of assessment.

	**PFT (n= 53)**		**Δ%**	**PAS (n= 55)**		**Δ%**

	Pre	Post		Pre	Post	
**9MIN**_(m)_	1316.9±318.7	1653.2±244^*^	25.5	1364.3±285.3	1384.3±288.1	1.5
**ULEP**_(cm)_	3.1±0.7	3.3±0.5^*^	6.5	2.9±0.7	3,0±0.7	3.4
**LLEP**_(cm)_	1.5±0.3	1.7±0.4^*^	13.3	1.5±0.4	1.7±0.6	13.3
**LMEabd**_(rep)_	30.5±10	38.9±10.6^*^	27.5	31.8±9.5	36.3±11.1^*^	14.2
**AGILst**_(s)_	6.7±0.7	6.7±1.8	0.0	6.9±0.6	6.6±0.8^*^	−4.3
**SP20m**_(s)_	3.9±0.57	3.7±0.4^*^	−5.1	4,0±0.5	3.9±.7^*^	−2.5
**FLEXw/b**_(cm)_	30.9±10.6	40.4±12^*^	30.7	32.7±13.3	39,0±15.1^*^	19.3

(*) significant difference between the pre and post-test (p<0.05);

PFT = physical fitness training; PAS = self-selected physical activity; 9MIN = cardio-respiratory (9 min); LMEabd = abdominal endurance; FLEXw/b = flexibility (sit-and-reach); SP20m = 20 m running; LLEP = horizontal jump test; ULEP = medicine ball throwing; AGILst = agility.
